# Impact of ureteric stent diameter on stent‐related symptoms and early outcomes after kidney transplantation: A randomised controlled trial

**DOI:** 10.1002/bco2.70166

**Published:** 2026-01-25

**Authors:** Ratchanon Wongtreeratanachai, Yada Phengsalae, Nuttapon Arpornsujaritkun, Surasak Kantachuvesiri, Kittinut Kijvikai, Kun Sirisopana, Wisoot Kongchareonsombat, Premsant Sangkum, Chinnakhet Ketsuwan

**Affiliations:** ^1^ Division of Urology, Department of Surgery, Faculty of Medicine Ramathibodi Hospital Mahidol University Bangkok Thailand; ^2^ Excellent Center for Organ Transplantation Ramathibodi Hospital Mahidol University Bangkok Thailand

**Keywords:** double‐J stents, kidney transplantation, stent‐related symptoms, ureteroneocystostomy, USSQ

## Abstract

**Objective:**

The aim of this study is to evaluate the impact of ureteric stent diameter on stent‐related symptoms and early urological complications in kidney transplant recipients.

**Patients and Methods:**

A single‐centre randomised controlled trial that enrolled 70 kidney transplant recipients to receive either a 4.8 Fr or 6 Fr ureteric stent allocated at a 1:1 ratio was conducted. Stent‐related symptoms and patient‐reported outcomes were assessed using the Ureteral Stent Symptom Questionnaire (USSQ) and a visual analogue scale (VAS) for pain. Early postoperative complications—including urinary leakage, ureteric obstruction and urinary tract infection (UTI)—were recorded.

**Results:**

The 4.8 Fr stents were associated with significantly fewer stent‐related symptoms and lower USSQ scores than 6 Fr stents (47.0 ± 4.5 vs. 53.9 ± 4.2; *p* < 0.001). Patients who received a 4.8 Fr stent experienced lower pain intensity than those who received a 6 Fr stent (VAS 1.4 ± 0.7 vs. 2.2 ± 0.8; *p* < 0.001). Rates of urinary leakage, ureteric obstruction and UTI were comparable between the two groups.

**Conclusion:**

The 4.8 Fr ureteric stents reduce stent‐related symptoms and postoperative pain while demonstrating a similar early safety profile to 6 Fr stents. These findings support the use of smaller‐calibre stents to improve postoperative comfort following kidney transplantation.

## 1 INTRODUCTION

Kidney transplantation (KT) is the most effective and cost‐efficient treatment for end‐stage kidney disease, offering superior quality of life and long‐term survival compared to dialysis.^1^, ^2^, ^3^ Nonetheless, early postoperative major urological complications, particularly ureteric stenosis and urinary leakage, remain clinically important. These complications typically originate during the ureteroneocystostomy and are often the result of technical factors or ureteric ischaemia, and they can lead to morbidity, prolonged hospitalisation, graft dysfunction or graft loss.

While routine prophylactic stenting across the ureteroneocystostomy significantly reduces these complications,^4^, ^5^ stents may cause morbidity, including urinary tract infections (UTIs), haematuria, migration, encrustation, fragmentation, pain and lower urinary tract symptoms. UTIs are especially concerning in these cases because of their potential impact on graft function.^6^ Therefore, any preventive benefits of routine prophylactic stenting must be balanced against the risk of stent‐related morbidity.

Although ureteric stenting is widely practiced, there is limited evidence directly comparing the effect of stent diameter in KT. Optimal stent sizes may lead to reduced symptoms and maintain surgical safety. This study compares early postoperative outcomes and patient‐reported symptoms between 4.8 Fr and 6 Fr ureteric stents in KT recipients.

## 2 PATIENTS AND METHODS

We conducted a prospective randomised controlled trial at the Faculty of Medicine, Ramathibodi Hospital. We enrolled 70 adult patients undergoing kidney transplantation between February 2021 and November 2021 for the trial. The study adhered to the 2010 Consolidated Standards of Reporting Trials (CONSORT) guidelines. Ethical approval was obtained from the Institutional Review Board of the Faculty of Medicine, Ramathibodi Hospital (COA. MURA2020/2006), and the trial was prospectively registered with the Thai Clinical Trials Registry (TCTR20210204005). Written informed consent was obtained from all participants prior to their enrolment.

Eligible participants were adults >18 years of age undergoing kidney transplantation. Individuals with a reconstructed urinary tract, bladder dysfunction requiring intermittent or continuous catheterisation, and donor kidneys with multiple ureters were excluded.

The participants were randomly assigned at a 1:1 ratio to receive either a 4.8 Fr or a 6 Fr ureteric stent at the time of transplantation. Randomisation was performed using a computer‐generated sequence with concealed allocation. A completed CONSORT checklist is provided in the Supporting Information.

Because of the intrinsic differences in stent size and physical characteristics, this study was conducted as an open‐label randomised controlled trial. While participants and treating clinicians were not blinded, outcome assessors, including personnel responsible for Ureteral Stent Symptom Questionnaire (USSQ) scoring and radiologists evaluating postoperative complications, were blinded to group allocation.

Immunosuppression was administered according to an institutional protocol. No adjunctive medications for stent‐related symptom relief, including anticholinergics or alpha‐blockers, were routinely prescribed during the study period. A standardised surgical technique was used for all procedures. Preoperative urinalysis and urine culture were performed as part of routine transplant evaluation. All patients were placed under general anaesthesia and routinely received prophylactic cefuroxime 1.5 g intravenously. After a graft was placed in the iliac fossa and vascular anastomoses were completed by a vascular surgeon, ureteroneocystostomy was performed by a urologist using the modified Lich–Gregoir anti‐reflux technique. Ureteric stents (4.8 Fr or 6 Fr, Percuflex, Boston Scientific, Natick, MA, USA) were inserted according to the group each patient belonged to. A 20 Fr Foley catheter was left indwelling and removed on postoperative day 7, while ureteric stents were removed on postoperative day 14.

The patients' baseline demographic and clinical data were recorded at enrolment. Patient‐reported symptoms were assessed using the visual analogue scale (VAS) for pain, documentation of stent‐related symptoms and a Thai‐translated version of the USSQ that was validated and translated by Watcharawittayakul et al.^7^ The USSQ is a multidimensional instrument designed to quantify the severity of stent‐related symptoms and their impact on quality of life across domains, including pain, urinary symptoms, general health, work performance and sexual matters. The USSQ and VAS assessments were completed the day before stent removal. Postoperative adverse events were recorded during hospitalisation and at the 3‐month follow‐up visit.

### 2.1 Outcomes

The primary outcomes were stent‐related complications and patient‐reported measures, including pain assessed using a VAS, visible haematuria requiring catheterisation with or without bladder irrigation, stent migration confirmed by ultrasonography or radiography, stent fragmentation, postoperative UTI, stent‐related urinary symptoms (e.g. discomfort, lower urinary tract symptoms, and mild visible haematuria) and domain and total scores. The VAS consisted of a 10‐cm horizontal scale anchored by ‘no pain’ and ‘worst imaginable pain’.”

UTIs were defined as follows: symptomatic UTI was defined as pyuria accompanied by a midstream urine (MSU) culture of >10^2^ CFU/ml, while asymptomatic bacteriuria was defined as pyuria with an MSU culture of >10^5^ CFU/ml.^8^

The secondary outcome was the incidence of early major urological complications within 3 months after transplantation, including ureteroneocystostomy anastomotic leakage or ureteric stenosis. Graft loss was defined as an estimated glomerular filtration rate (eGFR) of <15 ml/min/1.73 m^2^, return to dialysis or retransplantation.

### 2.2 Sample size

The sample size was calculated based on data from a previous study on ureteric stents.^8^ Using STATA version 14.1 (StataCorp, College Station, TX, USA), we estimated that detecting a difference in mean USSQ scores (54.3 vs 58.7) with standard deviations of 5.2 and 4.8, a two‐sided alpha of 0.01 and a 90% power would require at least 24 participants per group. To account for potential dropouts and to enhance statistical robustness, target enrolment was increased to 35 patients per group.

### 2.3 Statistical analysis

All statistical analyses were performed using STATA version 14.1 (StataCorp, College Station, TX, USA). Continuous variables were summarised as means ± standard deviations (SD), and categorical variables were presented as frequencies and percentages. Between‐group comparisons were conducted using independent *t*‐tests for continuous variables and Fisher's exact tests for categorical variables, as appropriate. A *p*‐value < 0.05 was considered statistically significant.

## 3 RESULTS

**FIGURE 1:**
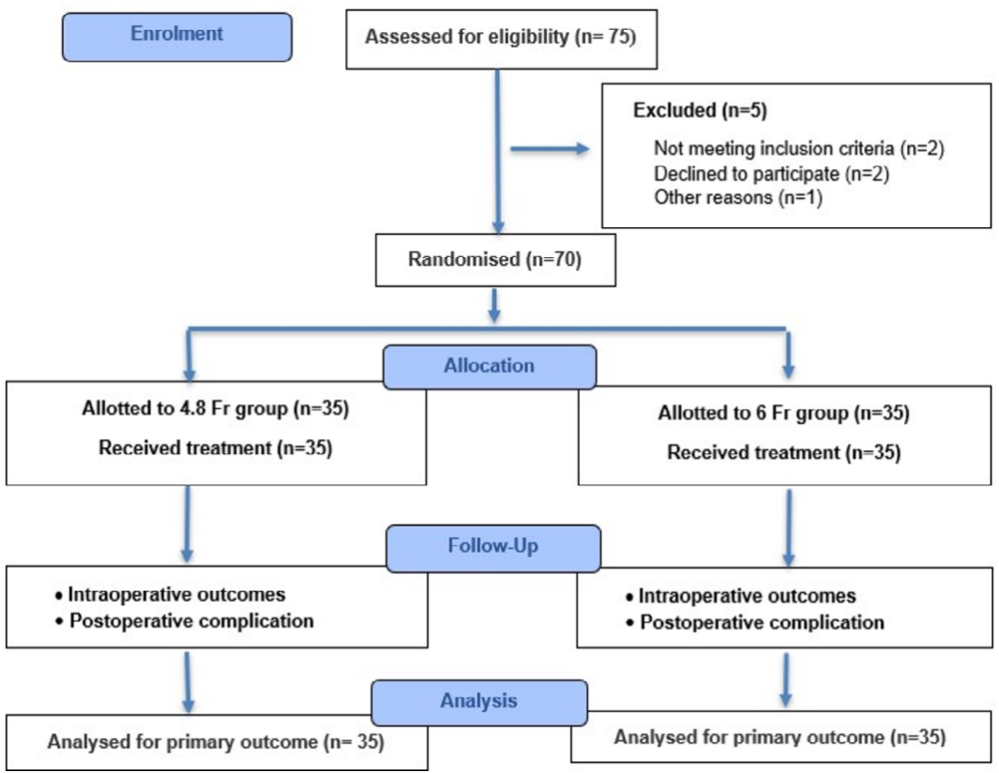
Flow of patients during the study.

A total of 70 kidney transplant recipients were enrolled, with 35 patients allocated to each arm (4.8 Fr stent group and 6 Fr stent group) (Figure 1). Baseline demographic and operative characteristics were comparable between the two groups (Table 1). Most kidney transplants were from deceased donors, with no significant difference in donor type between the two stent groups.

**TABLE 1:** Baseline characteristics and operative parameters.

Variable	4.8 Fr (*n* = 35)	6 Fr (*n* = 35)	*p*‐value
Age (years) (mean ± SD)	40.9 ± 8.8	42.0 ± 10.5	0.658
Gender: *n* (%)			
Male	14 (40.0)	14 (40.0)	1.000
Female	21 (60.0)	21 (60.0)	
Laterality: *n* (%)			
Left	17 (48.6)	17 (48.6)	1.000
Right	18 (51.4)	18 (51.4)	
BMI (kg/m^2^) (mean ± SD)	22.5 ± 3.7	22.3 ± 3.5	0.801
Donor type: *n* (%)			
Living donor	5 (14.3)	4 (11.4)	0.721
Deceased donor	30 (85.7)	31 (88.6)	
Previous renal transplant: *n* (%)	0 (0)	0 (0)	1.000
Diabetes mellitus: *n* (%)	4 (11.4)	5 (14.3)	0.721
Operative time (min) (mean ± SD)	310.6 ± 60.7	299.8 ± 33.9	0.365
Estimated blood loss (ml) (mean ± SD)	472.0 ± 336.6	422.9 ± 308.3	0.526
Blood transfusion requirement: *n* (%)	2 (5.7)	2 (5.7)	1.000
Length of hospital stay (days) (mean ± SD)	17.6 ± 16.5	15.2 ± 18.5	0.568

Abbreviation: BMI, body mass index.

In terms of stent‐related complications, postoperative UTIs occurred in 25.7% of patients in the 4.8 Fr group and 20.0% in the 6 Fr group (*p* = 0.569) (Table 2). Following ureteric stent removal, one case of asymptomatic bacteriuria was observed in each group, with no statistically significant difference between groups. Pain intensity (VAS), stent‐related symptoms and USSQ scores were all significantly lower in the 4.8 Fr group compared to those of the 6 Fr group (all *p* < 0.001). One episode of visible haematuria was observed in the 6 Fr group and was successfully managed with bladder irrigation. No cases of stent fragmentation or migration were noted in either group.

**TABLE 2:** Analysed outcome measures.

Variable	4.8 Fr (*n* = 35)	6 Fr (*n* = 35)	*p*‐value
Stent symptoms: *n* (%)	0 (0)	4 (11.4)	0.039*
USSQ score (mean ± SD)	47.0 ± 4.5	53.9 ± 4.2	<0.001*
VAS‐pain (mean ± SD)	1.4 ± 0.7	2.2 ± 0.8	<0.001*
UTI: *n* (%)	9 (25.7)	7 (20.0)	0.569
Haematuria: *n* (%)	0 (0)	1 (2.9)	0.314
Stent migration: *n* (%)	0 (0)	0	1.000
Fragmentation: *n* (%)	0 (0)	0	1.000
Ureteric leak: *n* (%)	2 (5.7)	1 (2.9)	0.555
Ureteric stenosis: *n* (%)	0 (0)	0 (0)	1.000
Graft failure: *n* (%)	0 (0)	0	1.000
Patient deaths: *n* (%)	0 (0)	0	1.000

Abbreviations: USSQ, ureteral stent symptom questionnaire; UTI, urinary tract infection; VAS, visual analogue scale.

* Statistically significant.

Regarding major urological complications, urinary leakage occurred in two patients (5.7%) in the 4.8 Fr group and in one patient (2.9%) in the 6 Fr group, indicating no statistically significant difference (*p* = 0.555). All affected patients required open surgical revision. No ureteric stenosis or graft failure occurred, and no patients died.

## 4 DISCUSSION

Ureteric stenting is routinely performed during kidney transplantations to reduce the likelihood of early anastomotic complications. However, stent‐related morbidity remains a concern, and studies directly comparing different stent diameters in transplant recipients have remained limited. In this randomised controlled trial, the use of a 4.8 Fr ureteric stent resulted in significantly fewer stent‐related symptoms, lower USSQ scores and reduced postoperative pain in patients compared to the use of a conventional 6 Fr stent and did not increase major urological complications. These findings indicate that stent diameter is an important determinant of postoperative tolerability.

Stent‐related symptoms, including discomfort, storage lower urinary tract symptoms and haematuria, were observed exclusively in the 6 Fr group. This aligns with established literature, which provides evidence of bladder irritation from the distal coil, ureteric smooth‐muscle spasms, and mechanical friction along the ureter. Our results are consistent with previous non‐transplant studies in which smaller‐calibre stents were found to improve patient comfort without increasing the risks of migration, obstruction or fragmentation.^9^, ^10^, ^11^ The present study extends this evidence to kidney transplantation, supporting the use of smaller‐calibre stents in this unique patient population.

Importantly, the smaller stent diameter in the 4.8 Fr group did not compromise safety. Rates of urinary leakage, ureteric obstruction and UTI were similar between the groups. This is consistent with prior evidence showing that UTI risk in transplant recipients is more influenced by stent dwell time and host factors, such as diabetes, female sex and immunosuppression, than by stent diameter.^12^, ^13^ Our findings therefore suggest that downsizing the stent does not increase infectious or anastomotic complications when used within a standardised 14‐day dwell period.

Previous transplant studies have primarily focussed on whether stents should be used and on defining optimal removal timing, with several studies demonstrating that the first two postoperative weeks carry the highest risk of anastomotic complications^14^, ^15^, ^16^, ^17^ because of stent use. While these studies highlight the importance of stenting itself, the question of optimal stent diameter remains unanswered. Our trial addresses this gap by demonstrating that a 4.8 Fr stent offers improved symptom burden with no adverse impact on early urological outcomes.

Overall, the findings support the use of smaller‐calibre ureteric stents as well‐tolerated and safe options in kidney transplantation. The 4.8 Fr stents may enhance patient comfort, reduce postoperative urinary symptoms and streamline recovery without compromising graft‐related outcomes. Larger multicentre trials with longer follow‐up are warranted to validate these findings and to refine stent selection and dwell‐time strategies in transplant practice.

This study has several limitations. First, the work's single‐centre design may limit the generalisability of its results to centres with different surgical techniques or perioperative protocols. Second, the sample size, although adequate for ensuring the statistical significance of USSQ outcomes, may not aid in the detection of very small differences in rare complications, such as ureteric strictures. Finally, the short‐term follow‐up period focussed on early postoperative outcomes, and long‐term effects on ureteric patency or graft function were not evaluated. Future multicentre studies with longer follow‐up would help validate these findings and further define optimal stenting strategies for kidney transplantation.

## 5 CONCLUSIONS

The 4.8 Fr ureteric stents significantly reduce stent‐related symptoms and postoperative pain while maintaining similar early rates of major urological complications as 6 Fr stents. These results support the routine use of smaller‐calibre stents to improve postoperative comfort in kidney transplant recipients. Further multicentre studies with longer follow‐up are warranted.

## AUTHOR CONTRIBUTIONS

**Ratchanon Wongtreeratanachai:** Conceptualization; methodology; data curation; investigation; writing—original draft; writing—review and editing. **Yada Phengsalae:** Formal analysis; data interpretation. **Nuttapon Arpornsujaritkun:** Investigation; data interpretation. **Surasak Kantachuvesiri:** Investigation; data interpretation. **Kittinut Kijvikai:** Data interpretation; validation. **Kun Sirisopana:** Data interpretation; validation. **Wisoot Kongchareonsombat:** Investigation; data interpretation. **Premsant Sangkum:** Data interpretation; validation; visualization; writing—review and editing. **Chinnakhet Ketsuwan:** Project administration; data interpretation; validation; visualization; supervision; writing—review and editing.

## CONFLICT OF INTEREST STATEMENT

The authors declare that they have no competing interests.
